# Sustainable Power Generation with an All-Silk Electronics-Based Yeast Wearable Biobattery

**DOI:** 10.1021/acsomega.5c00131

**Published:** 2025-03-20

**Authors:** Rita Policia, Ricardo Brito-Pereira, Carlos M. Costa, Senentxu Lanceros-Méndez, Frank N. Crespilho

**Affiliations:** a Physics Centre of Minho and Porto Universities (CF-UM-UP) and Laboratory of Physics for Materials and Emergent Technologies, LapMET, University of Minho, Braga 4710-057, Portugal; b Institute of Science and Innovation for Bio-Sustainability (IB-S), University of Minho, Braga 4710-053, Portugal; c BCMaterials, Basque Center for Materials, Applications and Nanostructures, UPV/EHU,Science Park, Leioa 48940, Spain; d IKERBASQUE, Basque Foundation for Science, Bilbao 48009, Spain; e São Carlos Institute of Chemistry, University of São Paulo (USP), São Carlos 13560-970, Brazil

## Abstract

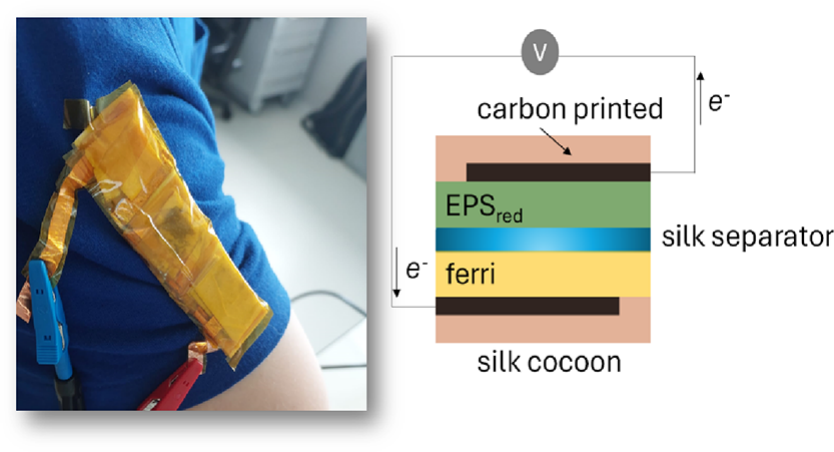

Transient electronics, designed to disintegrate in a controlled manner after their useful life, have been proposed as a solution to mitigate the ecological and health impacts of electronic waste (e-waste). Despite this innovative approach, which has seen significant application in biologically integrated sensors and therapeutic devices, it still results in the accumulation of different materials and nanomaterials for the powering systems often based on batteries, which themselves contribute to the e-waste problem. Here, we explore the use of the silk cocoon from *Bombyx mori* as a key component in the development of environmentally friendly all-silk electronics-based biobatteries. The approach focuses on employing *Saccharomyces cerevisiae* to generate electroactive extracellular polymeric substances, which serve as the anode material within the biobattery. The silk cocoon’s natural properties are utilized for the membrane in both anodic and cathodic compartments, with potassium ferricyanide embedded within the silk fibroin acting as the cathode. By coupling three modules in series, ohmic loss is minimized, preserving the voltages of each module. This setup allows a biobattery with discharge at a voltage over 1.1 V, demonstrating its potential to deliver stable and sufficient power for applications. The biobattery demonstrated a 95.2% utilization of recyclable materials for housing, membrane, and electrode components and a 95.6% utilization of biodegradable components for the electrolyte, offering a promising pathway for the advancement of eco-friendly energy storage solutions.

## 1 Introduction

Electronic waste (e-waste) is rapidly increasing, introducing significant toxic pollutants into the environment. Transient electronics, designed to disintegrate after use, has been proposed to mitigate the ecological and health impacts of e-waste.^1,2^ The goal is to reach fully degradable and/or recyclable electronics, where harmful components are avoided or, whenever present, recaptured and reused. Current efforts focus on recovering liquid–metal conductive traces or degrading single components, but a comprehensive system addressing all of the components would be more impactful. Additionally, manufacturing recyclable electronics at high temperatures poses high energy demands and environmentally harmful conditions, highlighting the need for more sustainable methods.^1−5^ Batteries are critical devices in this context as their production and disposal contribute significantly to e-waste. The accumulation of toxic materials waste from batteries adds to the overall e-waste problem.^3−5^ Furthermore, the manufacturing process for recyclable batteries often requires high temperatures, leading to increased energy consumption and environmental damage. Addressing these challenges is essential for developing more sustainable and eco-friendlier electronic solutions.

In this context, our study contributes to the development of eco-friendly biobatteries using silk-based printing technology. The yeast–silk printed biobatteries are designed to be fully biodegradable and recyclable, reducing the environmental footprint of disposable electronic devices. Printed circuit boards can be more environmentally friendly through the integration of sustainable materials and methods, particularly in the context of biobatteries.^2^ Utilizing biodegradable and renewable materials like silk fibroin for substrates and organic conductive inks for circuitry can significantly reduce the environmental impact. This aligns with the eco-friendly principles of biobatteries, which use biodegradable components derived from renewable sources. By combining these sustainable practices, the entire biobattery system promotes a circular economy, minimizes e-waste and supports the development of environmentally responsible electronic devices.

The silk cocoon of the silkworm, *Bombyx mori* (BM), stands as a remarkable example of a natural biomaterial boasting unique structural and biochemical properties.^6^ Composed primarily of fibroin and sericin proteins, silk’s fibroin, serving as the core structural protein, comprises long polypeptide chains abundant in amino acids like glycine, alanine, and serine.^7^ These chains form beta-sheet structures, endowing silk with its renowned mechanical strength and flexibility. Acting as a gumming protein, sericin envelops the fibroin fibers, acting as a glue and contributing to the integrity and protection of the silk fibers. Fibroin’s remarkable biocompatibility, biodegradability, and robust mechanical properties render it a versatile material for a myriad of applications, spanning from biomedical devices and tissue engineering to advanced materials science.^8,9^ Leveraging silk’s specific physical–chemical properties has led to innovative applications, such as the development of biobatteries^10^ and separator membranes for bioelectrochemical systems.^11,12^ Although the use of silk-derived materials as separators has been explored in previous studies of refs (11) and (12), structural control and its direct impact on electrochemical performance have not been previously detailed in the literature. Understanding how morphological properties influence separator performance is crucial for optimizing ion transport, minimizing ion crossover, and enhancing the overall stability and efficiency of biobatteries.

Here, we explore the use of the silk cocoon from BM as a key component in the fabrication of environmentally friendly biobatteries. Specifically, silk fibroin’s robustness and biocompatibility offer a fertile ground for the development of an efficient and sustainable biobattery when combined with electroactive extracellular polymeric substances (EPS),^13−19^ such as those derived from *Saccharomyces cerevisiae* (SC). Silk fibroin can function as a stable and biocompatible substrate for immobilizing EPS. The beta-sheet structure of fibroin provides an ideal environment for the adhesion and activity of EPS, ensuring effective electron transfer processes within the biobattery. Moreover, silk fibroin can be processed into films or composites, functioning as electrodes and separators in the biobattery. Its high processability allows the fabrication of silk-based separators with distinct morphologies, providing a means of optimizing the system’s stability and performance. Additionally, silk fibroin’s mechanical strength guarantees durability for carbon-printed electrical contacts, while its biocompatibility supports the viability of microbial communities, bolstering the overall efficiency of the bioelectrochemical system.^20^

The development of a fibroin-EPS biobattery offers several advantages over previously reported systems.^10^ Unlike traditional biobatteries that often rely on synthetic or non-biodegradable materials, with rigid architectures that limit their potential for integration into flexible and portable devices, the integration of biodegradable, biocompatible, and flexible all-silk bioelectronic platforms paves the way for reduced electronic waste and enhances adaptability to a wide range of wearable applications. Exploring the multifunctionality of silk fibroin, this adaptability makes it particularly suitable for use in devices that require lightweight, biocompatible, and efficient power sources, while its modular design enables easy scalability and customization based on the specific energy requirements of different applications.

## 2 Experimental Section

### Materials

Phosphate salts, specifically NaH_2_PO_4_·H_2_O (137.99 g mol^–1^, >98%) and Na_2_HPO_4_·7H_2_O (168.07 g mol^–1^, >98%), were obtained from Merck. Sodium hydroxide (NaOH, 39.997 g mol^–1^, 99%) and anhydrous d-glucose (99%) were also sourced from Merck. BM silkworm cocoons were supplied by APPACDM from Castelo Branco, Portugal. Formic acid (CH_2_O_2_, or FA), sodium carbonate (Na_2_CO_3_), and calcium chloride (CaCl_2_) were procured from Sigma-Aldrich. Carbon ink was obtained from Dupont (Delaware, USA). All aqueous solutions were prepared by using deionized water with a resistivity of 18 MΩ cm at 25 °C.

### Electrode Preparation on the BM Cocoon Substrate

**Figure 1:**
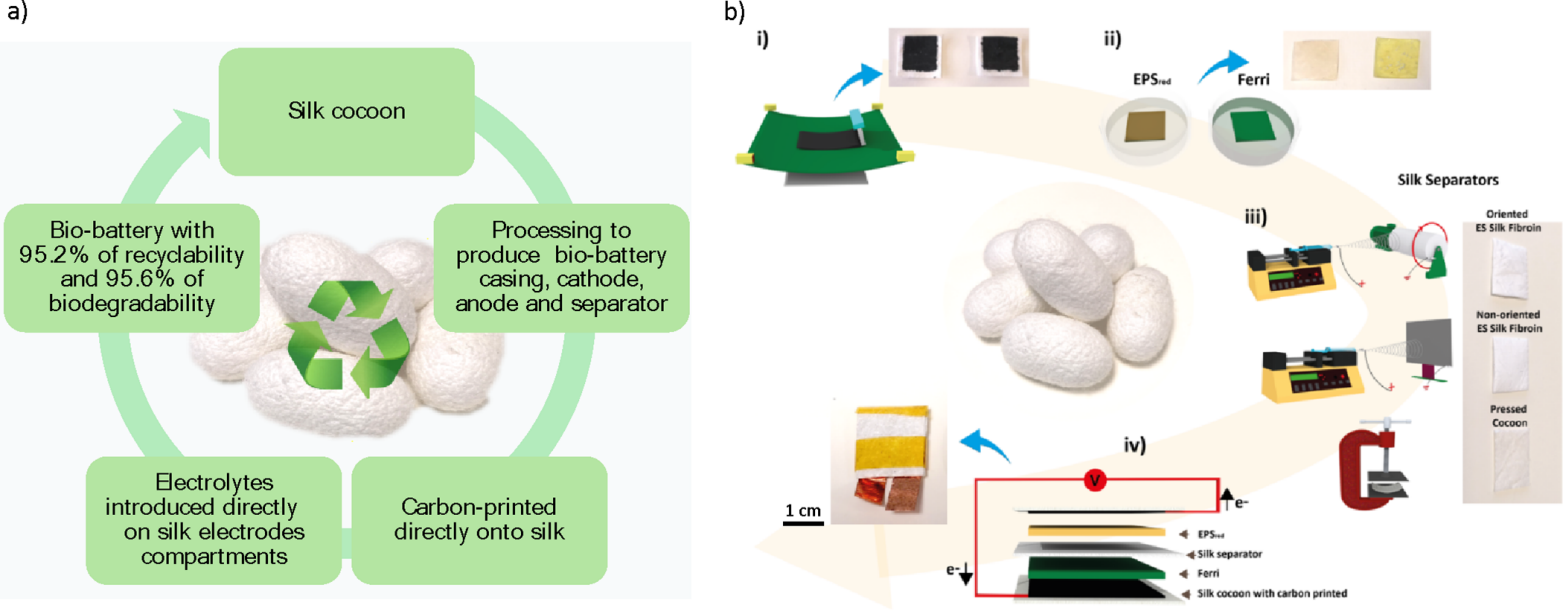
Biobattery assembly and bioelectronic printing. (a) Representation of the stages of preparation of all biobattery components starting from the silkworm cocoon, where the preparation of the bioelectrodes is systematically described, the obtaining of the anodic and cathodic compartments, and the description of biodegradability and recyclability as key components of the biobattery electronics. This closed cycle aligns with the concept of a circular economy and sustainability. (b) Schematic representation of the biobattery fabrication process. (i) Screen printing of carbon electrodes on a cocoon substrate. (ii) Anode and cathode fabrication by soaking fibroin films in EPS reduced and Ferri saturated solutions, respectively, on Petri dishes. (iii) Process of the three different biobattery separators used in this work: oriented and randomly oriented silk fibroin fibers fabricated by electrospinning and cocoon pressed separator using a mechanical press. (iv) Schematic of the biobattery architecture and photographic picture of the mounted battery.

BM cocoons were cleaned and sectioned into pieces with an approximate area of 2.25 cm^2^. These pieces were then compressed using a mechanical press (Model 4350.L Bench Top, Carver, Inc., USA) at a pressure of 5 tons for 3 h to reduce their thickness and create a flat surface suitable for printing purposes. Subsequently, quadrangular carbon electrodes with an area of 1 cm^2^ were deposited onto the flattened cocoon substrates using screen printing (DSTAR, model DX-305D) with a polyester mesh (120 wires) (Figure 1 and Figure S1). The printed samples were then cured in an oven (JP Selecta, Model 2000208) at 80 °C for 20 min.

### Preparation of Silk Fibroin Films

Silk fibroin (SF) was extracted from BM cocoons and purified following the method described in the Supporting Information. The extracted SF was then dissolved in formic acid (FA) at a 10:1 (v/w) ratio and homogenized using a magnetic stirrer. The resulting solution was cast into a Petri dish and left to stand in an airing chamber at room temperature for 24 h to facilitate solvent evaporation. The resulting films had a thickness of approximately 80 μm.

### Fabrication of Bioanode Material

SC was cultured by suspending 0.05 g mL^–1^ baker’s yeast in a solution of sterile phosphate buffer solution (0.10 mol L^–1^, pH 7.2), supplemented with 1.00 mol L^–1^ glucose. The yeast was incubated for 24 h at 40 °C under anaerobic conditions to promote growth and metabolic activity, producing EPS, suitable to be used as a bioanode active material. The resulting solution was then aliquoted into 30 mL sealed bottles and frozen at −80 °C overnight in preparation for the lyophilization process. SC/EPS frozen solutions were lyophilized for 3 days by using a Scanvac CoolSafe lyophilizer (LaboGene, CEB), yielding dehydrated EPS powder. Then, 10% (w/v) of EPS powder was dissolved in 50 μL of sodium phosphate solution (0.1 M). This solution was cast onto SF films (5 × 5 mm), facilitating the incorporation of the fungal exopolysaccharide into the SF matrix and forming the bioanode composite used to fabricate the biobattery.

### Fabrication of Biocathode Material

To produce the SF biocathode, 10% (w/v) hexacyanoferrate was dispersed in 0.1 M phosphate buffer solution. After that, 50 μL of the solution was deposited in the SF film (5 × 5 mm) to incorporate the hexacyanoferrate in the matrix, forming the biocathode material used to fabricate the biobattery (Figure 1b,ii).

### Fabrication of Silk-Based Separators

Cocoon separators were prepared using the same procedure as that described for the preparation of cocoon substrates. SF separators were fabricated through electrospinning. First, SF was dissolved in FA at an 8:1 v/w FA ratio. This solution was transferred to a 10 mL disposable syringe with a blunt steel needle (0.5 mm inner diameter) and placed in a syringe pump (New Era NE-1000). Electrospinning was performed by using a Glassman PS/FC30P04 high voltage power source set at 15 kV, with a flow rate of 0.5 mL/h. Non-oriented electrospun SF fibers were collected on a grounded static plate collector (20 cm × 15 cm), positioned 15 cm from the needle’s tip. Oriented electrospun SF fibers were produced using a grounded rotating drum collector set at 1500 rpm (Figure 1b,iii).

### Biobattery Assembly

The biocathode and bioanode materials were placed onto two separate carbon-printed electrodes, forming the bioelectrodes. The assembly of a biobattery involved three essential stages. First, the bioelectrodes were positioned with a separator membrane placed between the bioanode and the biocathode. This step ensures accurate alignment and spacing of the components within the battery (Figure 1b,iv). Second, the battery was sealed using Kapton adhesive tape for electrical and thermal insulation. Finally, the initial tests were conducted with the battery positioned on a fixed solid surface (Figure S3).

### Characterization

The surface morphology evaluation of the cocoon substrates, carbon electrodes, and silk separators was carried out by scanning electron microscopy (SEM) using a Carl Zeiss EVO 40 with an accelerating voltage of 20 kV. The samples were previously prepared with a conductive gold layer coating (Polaron, model SC502). Electrochemical measurements, including power curves, open circuit voltage (OCV), galvanostatic charge–discharge curves, polarization curves, and potentiostatic charge–discharge curves, were performed using a PalmSense potentiostat (PalmSense4). Each of these measurement protocols is explained in detail in Supporting Information. The biobattery’s weight was measured with a precision scale (Kern ADB), ranging from 240 mg for the SF electrospun separators to 300 mg for the cocoon separator, indicating differences in membrane composition or thickness.

## 3 Results and Discussion

Figure 1a,b illustrates the biobattery design employing yeast-derived EPS as the anode material and potassium ferricyanide as the cathode material (see also Figure S2). The anode is fashioned using a biofilm of EPS produced by SC yeast, well known for its conductive properties for efficient electron transfer.^14^ EPS produced by SC exhibit several key characteristics that make them suitable for use in anodes of biobatteries. These EPS are primarily composed of complex polysaccharides that form a robust and conductive biofilm, facilitating efficient electron transfer processes. Their molecular structure includes repeating units of glucose and mannose, contributing to their high electrical conductivity and stability. EPS possess excellent adhesive properties, enhancing the overall structural integrity of the biofilm.^13−19^ The cathode material, potassium ferricyanide (ferri), although it is not biodegradable, is selected for its well-characterized and efficient redox stability and compatibility with the biobattery environment.

**Figure 2:**
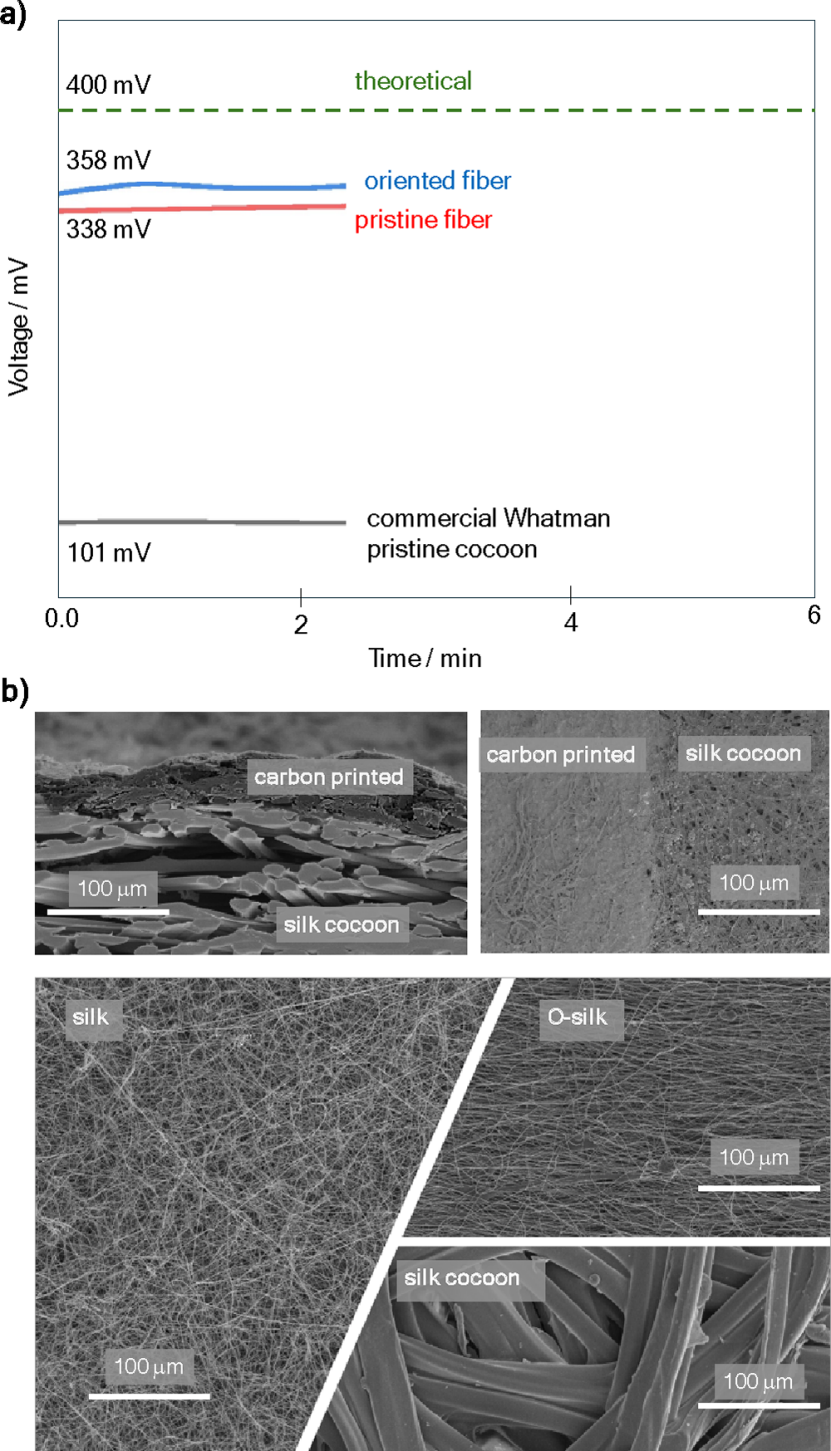
Biobattery membrane performance and morphological features. (a) Voltage with different membranes, using oriented fiber silk (O-Silk), pristine fiber (non-oriented), commercial membrane, and pristine cocoon separator (overlaid to the commercial one). (b) SEM images for silk cocoon, carbon-printed silk cocoon, non-oriented fiber silk separator, and oriented fiber silk separator (O-Silk).

Four types of separators were evaluated, one of which is commercial (i), commonly used in battery tests (Whatman), and the other three were prepared from the material of the silk cocoon itself: (ii) the cocoon being pressed in its natural state (pristine cocoon), (iii) the silk fibers extracted and then deposited randomly by electrospinning (pristine), and (iv) silk fibers oriented by electrospinning (O-Silk). Upon assembly, the biobattery using O-Silk presented an initial open circuit voltage (OCV) of 358 mV (Figure 2), very close to the theoretical value (400 mV), indicative of the reliable electrochemical stability of its components. This value is 20 mV higher compared to randomly (pristine) silk fibers and significantly higher than when a commercial separator and pristine cocoon are used (Figure 2a and Figure S4). Furthermore, for silk fibers and commercial separators, electrolyte crossover was observed after a few minutes of operation. The SEM images show that the O-Silk separator exhibits a high orientation of the fibers (Figure 2b), whereas the non-oriented silk (pristine fiber) shows fiber entanglement. Both the O-Silk and non-oriented silk separators exhibit a uniform fiber diameter distribution of approximately 1 μm. The silk cocoon, on the other hand, consists of fibrillar structures with fiber diameters approximately 20 times larger than those of silk separators. This larger fiber size increases interfiber spacing, resulting in a less dense network with wider gaps between fibers, facilitating ion crossover and compromising the separator’s effectiveness. Between the two silk separators, O-Silk demonstrates superior performance, displaying a higher OCP even after several cycles (Figure S5). This can be attributed to its highly aligned fiber structure, which minimizes the distance between fibers, creating a compact fibrillar network without voids that effectively prevents crossover. Given these advantages, further optimizations of the biobattery will be carried on using the O-Silk separator.

Given that O-Silk demonstrated a higher OCP even after several cycles (Figure S5), from now on, we will particularly focus on the optimizations of the biobattery using the O-Silk separator. Although silk cocoon does not perform well as a separator (Figure S5a) due to its highly compacted structure (Figure 2b), it proves to be an excellent material for sealing the entire battery. Therefore, it was used in the manufacturing of the biobattery capsules, creating an all-silk system based on electronics, separator, and electrolyte compartment. As we shall show in the following, the battery can be also incorporated into fabrics. This is possible because it is flexible, and its operation is not hindered by movement, twisting, or bending.

**Figure 3:**
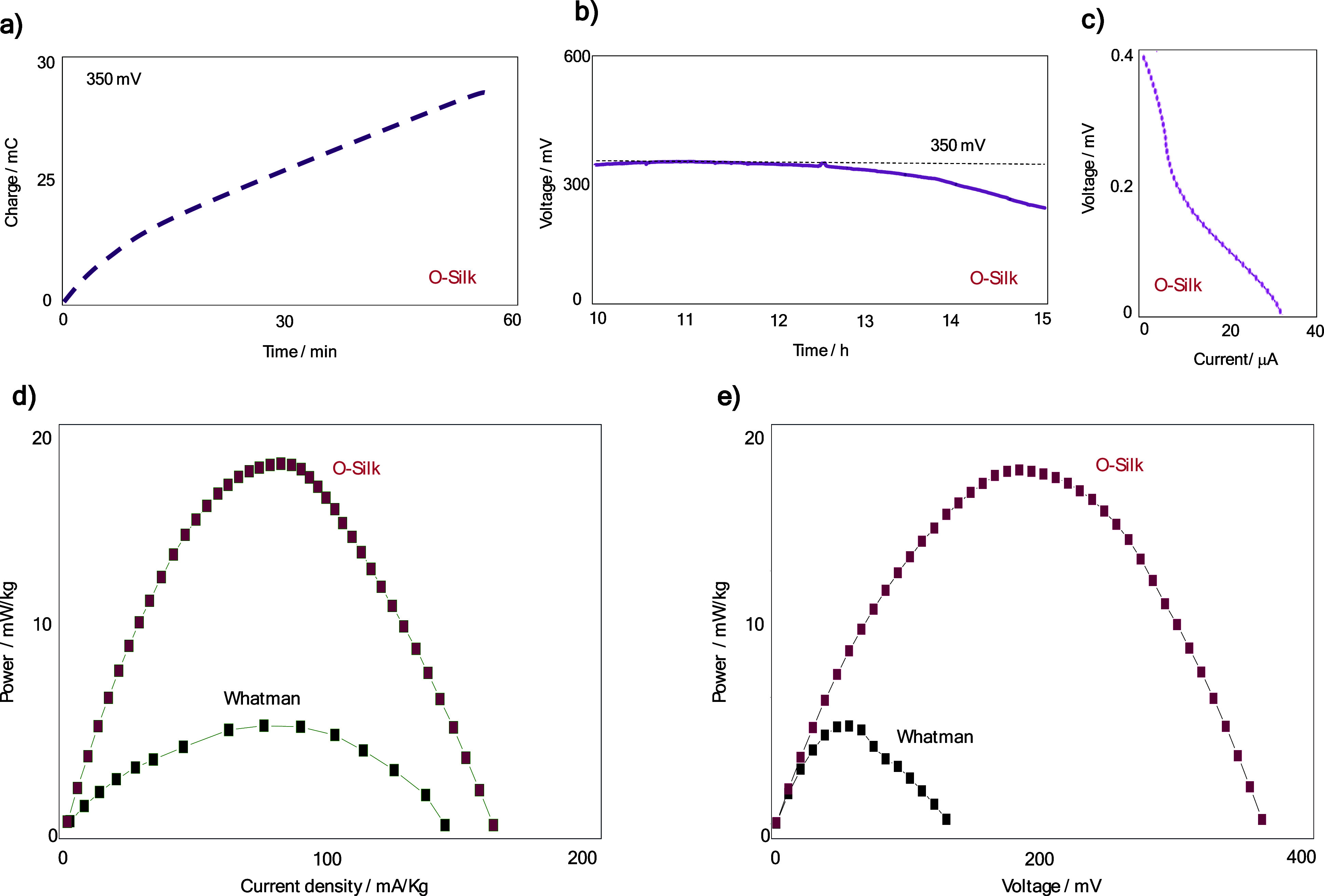
Biobattery performance.(a) Charge curve for the biobattery. (b) Discharge curve at 350 mV with a drainage current of 0.1 μA. (c) Current versus voltage for a single module biobattery. (d) Power curve as a function of current for the biobattery operating with an O-Silk separator compared to the commercial Whatman separator. (e) Power curve as a function of voltage for the biobattery operating with an O-Silk separator compared to the commercial separator.

Following the assembly process, a range of measurements are taken to evaluate the performance of the biobattery in different configurations (Figures S6–S14). OCV measurements were conducted using oriented fibroin fibers as the battery’s separator for different anodes and for three batteries in series using EPS as the anode (Figure S6). A comparison of polarization curves between the Whatman membrane and non-oriented fibroin fibers showed differences in performance, with polarization curves provided for both the Whatman membrane (Figure S7a) and non-oriented fibroin fibers (Figure S7b). Power curves using the Whatman membrane demonstrated the power curve dependence on current density and voltage for biobatteries (Figure S8a,b). Similarly, power curves using non-oriented fibroin fibers as separators highlighted the power curve dependence on current density and voltage (Figure S9a,b). When oriented silk fibroin fibers were used as separators, the power curve dependence on current density and voltage was also investigated (Figure S10a,b). Galvanostatic charge–discharge curves were recorded at different currents using pressed cocoon as the separator, with applied constant currents (Figure S11). Voltage constant charge–discharge cycles were also performed using randomly oriented silk fibroin fibers as separators, applying 1 and −1 V (Figure S12). Chronocoulometric measurements showed the charge curve of the biobattery using pressed cocoon (Figure S13a) and oriented silk fibroin fibers as separators (Figure S13b). Finally, cyclic voltammetry measurements were conducted on the biobattery (Figure S14). Based on these experiments, we present here the most suitable experimental conditions, which are summarized in Figure 3.

Current density and specific energy were assessed under varied load conditions. For instance, when the biobattery is charged at 1 V (Figure 3a), it can reach 30 mL in approximately 1 h. When discharged with 1 μA, it can achieve a lifetime of up to 13 h (Figure 3b), maintaining stable performance without significant degradation in voltage or current output. This operational discharge has set a record in the literature, surpassing the previous record of 7 h achieved with the self-gelling quinone-based wearable microbattery.^21^ While the former battery operated at 0.89 V and was alkaline, the yeast–silk biobattery operates at 358 mV and lasts approximately 13 h. It also has the added benefits of having a near-neutral pH of around 7 and EPS being completely biodegradable.

While the presented results are promising, it is essential to explore potential avenues for further enhancing the performance of this biobattery. One issue that can be raised is that, once the EPS in the anode compartment is saturated, it is theoretically impossible to increase the current output of the biobattery without altering the thickness of the anodic compartment. Additionally, since the cathode is saturated with ferricyanide, we can infer that the limiting half-reaction is due to the quantity of EPS in the anode. To test this hypothesis, we conducted an experiment using a hybrid carbon electrode combined with EPS and metallic copper. The goal was to evaluate whether more charges can be introduced into the anode without modifying the composition of the EPS. The same biobattery was tested but with the addition of metallic copper combined with the anode. If the anode limits the battery’s charge, then the presence of Cu, which will be converted to Cu^1+^, will indicate an increase in total voltage and current. The results show that when Cu is incorporated, the battery life extends to 20 h with a charge drain of 1 μA (Figures S15 and S16). Metallic copper (Cu) oxidizes to cuprous ions (Cu^+^). The OCV jumps from 350 to 500 mV, indicating that EPS is the limiting active material. This reveals that the energy output of the biobattery can be regulated in terms of the bioanode.

**Figure 4:**
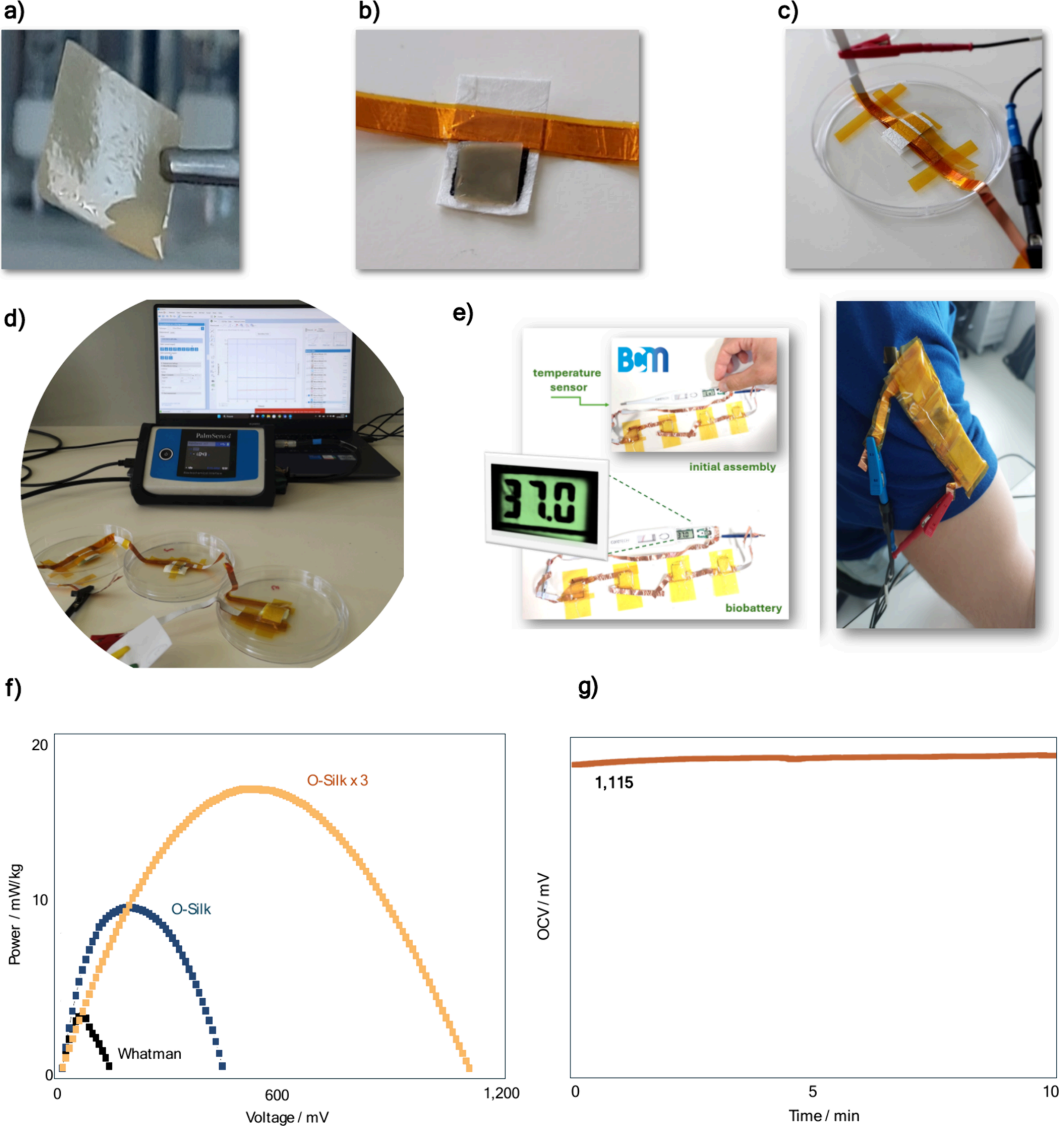
From lab bench to practical application prototype.(a) EPS with the silk membrane being pinched to be placed in the anodic compartment. (b) Positioning of the anodic membrane containing EPS over the carbon electrode previously printed on the silkworm cocoon. (c) Complete closure of the biobattery with the cathodic compartment on top of a Petri dish.(d) Integration of three biobattery modules in series for the measurement of electrochemical parameters such as the OCV and charge and discharge. (e) Visually exemplifying an application of a biobattery arrangement, in this case, the biobatteries are connected in a device for measuring body temperature (digital thermometer) and incorporated into fabric modules. (f) Power curve comparing a single module with a commercial separator, a single module with a separator prepared with oriented silk fibers, and three modules connected in series. The power curve demonstrates the assembly’s capacity, reaching nearly 20 mW per kg, and highlights the clear difference from a primary single module. The series coupling of the three modules results in minimal ohmic loss with the voltages of the independent modules being effectively preserved. (g) Measurements of the OCV of a battery containing three wearable modules incorporated into the fabric of a shirt.

The transition from lab bench to practical application prototype of biobatteries involves several critical steps, each significant from an electrochemical perspective. First, the EPS with a silk membrane is pinched to be placed in the anodic compartment (Figure 4a). The biocompatibility and excellent mechanical properties of the silk membrane make it an ideal support matrix for EPS, ensuring robust contact with the electrode surfaces. This step maximizes the anodic reaction surface area, enhancing electron transfer rates and overall cell efficiency. Next, the anodic membrane containing EPS is positioned over a carbon electrode printed on a silkworm cocoon (Figure 4b). This use of a carbon electrode leverages the high surface area and conductive properties of carbon combined with the natural, renewable substrate of the silkworm cocoon. This setup facilitates efficient electron flow and contributes to the sustainability of the biobattery design. The complete closure of the biobattery, with the cathodic compartment placed on top of a Petri dish, is shown in Figure 4c.

To assess the scalability capability, the stacking of batteries in series was evaluated. We conducted a sequence of experiments to evaluate the performance of three biobatteries connected in series. The electrochemical measurements of these biobatteries were recorded (Figure S17a), and the corresponding electrical circuit connected to a voltmeter is illustrated (Figure S17b). We also plotted the polarization curve behavior (Figure S18), the dependence of power on current density and applied voltage (Figure S19), and charge and discharge current of 10 μA, highlighting their performance over time (Figure S20). The integration of three biobattery modules in series is shown in Figure 4d. As expected, series integration increases the overall voltage output. The purpose of the biobattery arrangement is visually exemplified in Figure 4e. Here, the biobatteries are connected in a device for measuring body temperature (digital thermometer Caretech TS-101) and are incorporated into fabric modules. We used this temperature device because it is water-resistant, ensuring reliable performance in various environments. The device includes an audible alert feature for convenience. It measures temperatures in the range of 21–42.9 °C, suitable for many applications. Additionally, the device can memorize the last measurement taken, allowing for quick reference and continuity in monitoring. These features make it perfect for integration into a wearable biobattery, where consistent and reliable temperature monitoring is crucial. This integration into fabric highlights the flexibility and adaptability of the biobattery system for wearable electronics. Power curve comparison between a single module with a commercial separator, a single module with a separator prepared with O-Silk fibers, and three modules connected in series is shown in Figure 4f. The power curve demonstrates the assembly’s capacity, reaching nearly 20 mW per kg. The series coupling of three modules results in minimal ohmic loss, effectively preserving the voltages of the independent modules. This comparison emphasizes the importance of the separator material and module configuration in optimizing power density and minimizing internal resistance. Finally, measurements of the OCV of a battery containing three wearable modules incorporated into the fabric of a shirt are presented (Figure 4e). This practical implementation shows the feasibility of integrating biobatteries into everyday clothing. With this new configuration, the biobattery can achieve an OCV of 1.115 V. The OCV measurements demonstrate stable electrochemical performance of the wearable modules, confirming their potential for reliable energy supply in wearable electronics.

The significant increase in the biobattery’s duration is advantageous for long-term, low-consumption devices. With a near-neutral pH, it aligns well with green chemistry and sustainable development principles. The biobattery design leverages the natural properties of the silk cocoon (seeTable 1). The EPS-based anode exhibits excellent conductivity, while the cathode uses potassium ferricyanide for its redox stability. A separator made from sericin-enriched silk ensures effective ion transport while preventing ion crossover. These silk fibroin-based biobatteries stand out for their environmental sustainability, using biodegradable and renewable materials, unlike traditional batteries that rely on nonrenewable resources and contain toxic substances. Challenges for further development and improvement include exploring biodegradable alternatives to replace potassium ferricyanide, such as organic quinones, to enhance the biobattery’s biodegradability. Furthermore, achieving EPS saturation in the anode, which limits current output without altering the thickness of the fibroin film, remains a key focus. Optimizing EPS immobilization and exploring alternative configurations are essential for enhancing the electron transfer efficiency. To address these challenges, we engineered electrodes to couple three modules, maintaining the flexibility. This configuration achieved significant scalability potential, reaching 1.115 V when charged at 6 mC. The biobattery’s maximum power density approached nearly 20 mW kg^–1^, operating at 600 mV at maximum power. The stable discharge curve at 1 μA further demonstrates its potential. Silk fibroin’s mechanical durability and ion-selective properties significantly boost the biobattery’s efficiency and longevity. The biodegradability rate of the final configuration was performed utilizing 95.2% recyclable materials and achieved 95.6% biodegradability for the electrolyte (see the Supporting Information), highlighting its potential as an eco-friendly energy storage solution.

**Table 1:** Comparison of General Transient Electronics and Silk Fibroin-Based Biobattery Characteristics

parameter	transient electronics	yeast–silk biobatteries
materials	silicon-based materials, toxic chemicals	silk cocoon, *S. cerevisiae* EPS, ferricyanide
environmental impact	low recyclability	95.2% recyclable materials, 95.6% biodegradable components
anode material	various materials	EPS from *S. cerevisiae*
cathode material	various materials	potassium ferricyanide embedded in silk fibroin
separator	various synthetic materials	natural silk with sericin
open circuit voltage (OCV)	varies	0. 358 or 1.115 V (3× in series)
current density	varies	5–10 mA cm^–2^ (with O-Silk)
specific energy	varies	150–250 Wh kg^–1^
operational longevity	minutes to hours	13 h (22 h with the hybrid electrode)
maximum power density	varies	0.5–1 mW cm^–2^
environmental compatibility	can be harmful	near-neutral pH (∼7), fully biodegradable
scalability	commercial	promising with modular design and flexible nature
flexibility and integration	limited	can be integrated into fabrics, flexible
charge process	minutes to hours	25 mC cm^–2^ within 20 min (with O-Silk)
max current output	varies	38 μA (with O-Silk)
voltage stability	varies	stable discharge at 1.115 V
biocompatibility	limited	high, ideal for biomedical and wearable applications

## 4 Conclusions

A silk-based biobattery was developed, harnessing the biocompatibility and mechanical strength of silk fibroin. By integration of carbon-printed electrodes with EPS and utilization of the natural properties of silk cocoons, the design enhances durability and functionality. The biobattery, featuring a potassium ferricyanide cathode and a silk fibroin separator, demonstrates significant environmental sustainability and biocompatibility, ideal for medical devices and wearable electronics. Furthermore, the comparison between silk-based materials with different morphological properties and their direct impact on electrochemical performance provides an understanding of how separator architecture influences ion transport and overall biobattery stability. This study highlights the role of fiber orientation and diameter size in separator performance, demonstrating that the small fiber diameter (≈1 μm) and highly aligned fiber structure of the O-Silk separator minimize ion crossover and enhance biobattery efficiency.

Despite challenges in current density and active site quantification, the study achieves promising scalability, with three coupled modules showing stable and reliable performance, making it a suitable solution for low-power applications, such as biosensors, health monitors, and portable electronic devices. The silk fibroin-EPS biobattery represents a significant step forward in the development of sustainable, efficient, and adaptable biobatteries. The integration of an all-silk platform, the use of yeast-derived EPS for enhanced electron transfer, and the flexible, modular design for wearable applications make it a promising alternative to traditional biobased power sources and non-biodegradable energy storage devices.
