# Supplementary material for: Sustainable Power Generation with an All-Silk Electronics-Based Yeast Wearable Biobattery
Source: ACS Omega. 2025 Mar 20;10(12):12522–9. doi: 10.1021/acsomega.5c00131 (PMC11966269; doi:10.1021/acsomega.5c00131)
Supplement: Supplementary file 1 — ao5c00131_si_001.pdf [file ao5c00131_si_001.pdf]

Supporting Information

Sustainable Power Generation with All-Silk Electronics-Based Yeast Wearable Biobattery

Rita Policia[a,b], Ricardo Brito-Pereira[a,c], Carlos M. Costa[a], Senentxu Lanceros-Méndez[a,c,d*], Frank N. Crespilho[e*]

[a] *Physics Centre of Minho and Porto Universities (CF-UM-UP) and Laboratory of Physics for Materials and Emergent Technologies, LapMET, University of Minho, 4710-057 Braga, Portugal.*
[b] *Institute of Science and Innovation for Bio-Sustainability (IB-S), University of Minho, 4710-053, Braga, Portugal.*
[c] *BCMaterials, Basque Center for Materials, Applications and Nanostructures, UPV/EHU Science Park, 48940 Leioa, Spain.*
[d] *IKERBASQUE, Basque Foundation for Science, Bilbao, 48009, Spain.*
[e] *São Carlos Institute of Chemistry, University of São Paulo (USP), 13560-970, São Carlos, Brazil.*

*Email: senentxu.lanceros@bcmaterials.net, frankcrespilho@iqsc.usp.br

EXPERIMENTAL

Silk Fibroin Extraction and Purification Method. Silk fibroin (SF) was extracted from *Bombyx mori* (BM) cocoons using a soap degumming technique. The cocoons were cut into pieces (1 cm^2) and boiled in a 0.05% Na_2CO_3 solution for 30 minutes with a silk-to-water ratio of 1:40 w/v. The degummed silk fibroin fibers were thoroughly rinsed with distilled water and air-dried at room temperature for 24 hours. Subsequently, the fibers were dissolved in a 0.17 mol L^{-1} FA/$CaCl_2$ solution with a 12:1 w/v FA ratio. This solution was centrifuged at 6000 rpm for 10 minutes to remove impurities. The resulting SF/FA/$CaCl_2$ solution was spread onto a Petri dish and allowed to dry at room temperature for 24 hours to facilitate FA evaporation. The material was then immersed in distilled water for 48 hours to remove $CaCl_2$ by diffusion, yielding a brittle, whitish solid silk fibroin.

Open Circuit Voltage (OCV) Measurement. To establish the baseline performance of the biobattery, open circuit voltage (OCV) measurements were conducted. The setup involved connecting the biobattery to a potentiostat without any external load. Voltage readings between the electrodes were recorded over several hours. Stable OCV values over time indicated good electrode material stability and minimal internal short-circuiting, validating the initial assembly quality of the biobattery.

Galvanostatic Charge-Discharge Measurements. The biobatteries underwent galvanostatic charge-discharge tests to evaluate their capacity, energy density, and cycle stability. The setup involved connecting the biobattery to the potentiostat and setting a constant current. Charging occurred until a predefined voltage limit was reached, followed by discharging to a lower voltage limit. Voltage vs. time curves for both charge and discharge cycles were plotted to calculate specific capacity (mA h kg^{-1}) and energy density (Wh kg^{-1}). Repeated cycles assessed the cycle stability, monitoring any capacity fade, thereby determining the biobattery's practical energy storage and delivery capabilities.

Cyclic Voltammetry (CV). Cyclic Voltammetry (CV) provided insights into the electrochemical properties of the biobattery's electrodes, such as redox processes, reaction kinetics, and the reversibility of electrochemical reactions. The biobattery was connected to the potentiostat with a set scan rate, and the potential of the working electrode was linearly swept between two limits. Current responses were recorded, and current vs. voltage (I-V) curves were plotted to identify redox peaks.

Potentiostatic Charge-Discharge Measurements. Potentiostatic charge-discharge tests involved applying a constant voltage to the biobattery and measuring the resulting current over time. The biobattery was connected to the potentiostat with a desired voltage set. Constant voltage was applied, and the current response over time was recorded, followed by reversing the voltage polarity for the discharge phase. Current vs. time curves were plotted to analyze the charging and discharging profiles. The charge-discharge performance was evaluated under different current values, focusing on low currents (1 μA and 2 μA) and charge curves at higher currents to assess performance under varied

conditions. Experiments included variation of voltage during charging vs. time with different current values, long-term stability of OCV over 2 hours, and current density vs. voltage curves with different amounts of EPS in the anodic compartment. OCV measurements were taken after specific interventions, such as adding 300 µL of EPS and completing 6 charge-discharge cycles. Potentiostatic charge-discharge experiments at +1.0 V were conducted, and the resulting current vs. time data was analyzed. During quasi-steady state polarization after a 200-second charge-discharge cycle, the charge obtained was used to generate a power curve, contributing to the performance characterization of the biobattery.

Polarization Curves. Polarization curves were used to study the relationship between current density and voltage, providing insights into the overpotential and kinetic limitations of electrochemical reactions. The biobattery was connected to the potentiostat, and current density was incrementally varied while measuring the corresponding voltage. Steady-state voltage for each current density was recorded, and current density vs. voltage curves were plotted to identify regions corresponding to activation polarization, ohmic resistance, and concentration polarization. The overpotential and limiting current density were analyzed to identify kinetic and transport limitations, guiding improvements in electrode design and materials.

Long-term Stability Testing. Long-term stability tests were conducted to assess the durability and reliability of the biobattery over extended periods. The biobattery underwent repeated charge-discharge cycles, with parameters such as capacity, voltage profile, and internal resistance monitored.

Power Density and Energy Density Measurements. Power density and energy density are critical metrics for evaluating the biobattery's performance. Charge-discharge tests at different current densities were performed, and voltage and current were measured during these tests. Power density (W/kg) was calculated from the product of voltage and current divided by the biobattery's weight, and energy density (Wh/kg) was calculated from the integral of voltage over the charge-discharge cycle divided by the biobattery's weight.

Biobattery Scalability. This study addresses the scalability of silk-printed yeast biobatteries, focusing on overcoming power limitations to enhance performance for long-term applications in low-consumption devices while ensuring environmental sustainability. Similar to the single module system, a silk fibroin solution was extracted, prepared, cast, and dried to form films of consistent thickness. Yeast cells were cultured to produce EPS, which was harvested and purified for use in the biobattery. Electrodes were engineered to optimize coupling with EPS, ensuring maximum utilization of flavin sites without altering the film thickness. Individual biobattery units were assembled by integrating the silk fibroin film, and EPS with the modified electrodes. Three biobattery modules were sealed in parallel using Kapton tape. The biobattery was connected to a potentiostat without an external load to measure the OCV, and voltage readings were recorded over time to establish stability. Galvanostatic charge-discharge tests were conducted by performing charge-discharge cycles at various current densities, and voltage vs. time curves were plotted to determine specific capacity and energy density.

CV was performed to analyze redox processes and reaction kinetics by sweeping potential between set limits and recording current response. The performance of the three coupled biobattery modules was evaluated by measuring voltage, current, and power density during charge-discharge cycles. Power density (W/kg) and energy density (Wh/kg) were calculated from the charge-discharge data. Long-term stability tests involved repeating charge-discharge cycles over extended periods to monitor capacity retention and performance degradation. The pH of the biobattery electrolyte was measured to ensure near-neutral conditions. The voltage achieved (1.115 V) when charged at 6 mC. Discharge stability at 1 mA was maintained at 1.115 V. The maximum power density approached mW/kg, with the biobattery operating stably at 600 mV at maximum power. Scalability potential was demonstrated by integrating the biobattery into fabric structures.

Recyclability Rate (RR) Calculation. The RR of the biobatteries was calculated by evaluating the proportion of materials that could be recovered and reused after the biobattery had reached the end of its life. The process involved:

1. Identifying recyclable components such as metals, plastics, and other recoverable materials.
2. Determining the mass of each recyclable component.
3. Summing up the mass of all recyclable components.
4. Measuring the total mass of the biobattery.
5. Calculating the recyclability rate using the formula:

$$RR = \frac{mass\ of\ recyclable\ materials}{total\ mass\ of\ bio{-}battery} \times 100 \qquad \text{(S1)}$$

Biodegradability Rate (BR) Calculation. The BR was calculated to determine the percentage of the biobattery's materials that could decompose naturally by biological processes. The steps involved:

1. Identifying biodegradable components such as organic materials and natural polymers.
2. Measuring the mass of each biodegradable component.
3. Summing the mass of all biodegradable components.
4. Measuring the total mass of the biobattery.
5. Calculating the biodegradability rate using the formula:

$$BR = \frac{mass\ of\ biodegradable\ materials}{mass\ of\ bio{-}battery} \times 100 \qquad \text{(S2)}$$

Silk biobattery BR calculation

In order to calculate the amount of biodegradable materials present in the silk biobatteries, the different components of these were weighed.

Non degradable components:
- Weight of two carbon electrodes (area = 1 cm^2) = 2 x 2.9 = 5.8 mg
- Weight of 0.1M sodium phosphate in 100 μl solution = 3.2 mg
- Weight of hexacyanoferrate in 50 μl solution = 5 mg

Degradable components:
Weight of EPS = 5 mg
Weight of two BM cocoon electrodes = 2 x 71 mg = 142 mg
Weight of the silk separators:
O - Silk = 15 mg
Pristine fiber = 14 mg
Silk cocoon = 74 mg
Weight silk biocathode (BC) and bioanode (BA):
BC = 55 mg (of which 0.8 mg is sodium phosphate) = 54.2 mg
BA = 58 mg (of which 5 mg is hexacyanoferrate and 0.8 mg is sodium phosphate) = 52.2 mg

<u>BR of biobattery with O-Silk separator</u>

$$BR = \frac{(BM\ cocoon\ electrodes)+(O-Silk\ Separator)+(Silk\ Fibroin\ (BA\ and\ BC))}{total\ mass\ of\ the\ bio-battery} \times 100 \quad (S3)$$

$$= \frac{(61\ \times\ 2\ )+(30)+(54.2+\ 52.2\ )}{271} \times 100 = 95{,}3\ \%$$

<u>BR of biobattery with pristine fiber separator</u>

$$BR = \frac{(BM\ cocoon\ electrodes)+(Pristine\ fiber\ \ separator)+(Silk\ fibroin\ (BA\ and\ BC))}{total\ mass\ of\ the\ bio-battery} \times 100 \quad (S4)$$

$$= \frac{(61\ \text{x}\ 2\ )+(29)+(54.2+\ 52.2\ )}{270} \times 100 = 95{,}2\ \%$$

<u>BR of biobattery with pristine cocoon separator</u>

$$BR = \frac{(BM\ cocoon\ electrodes)+(Pristine\ cocoon\ separator)+(Silk\ Fibroin\ (BA\ and\ BC))}{total\ mass\ of\ the\ bio-battery} \times 100 \quad (S5)$$

$$= \frac{(61\ \text{x}\ 2\ )+(63)+(54.2+\ 52.2\ )}{305} \times 100 = 95{,}6\ \%$$

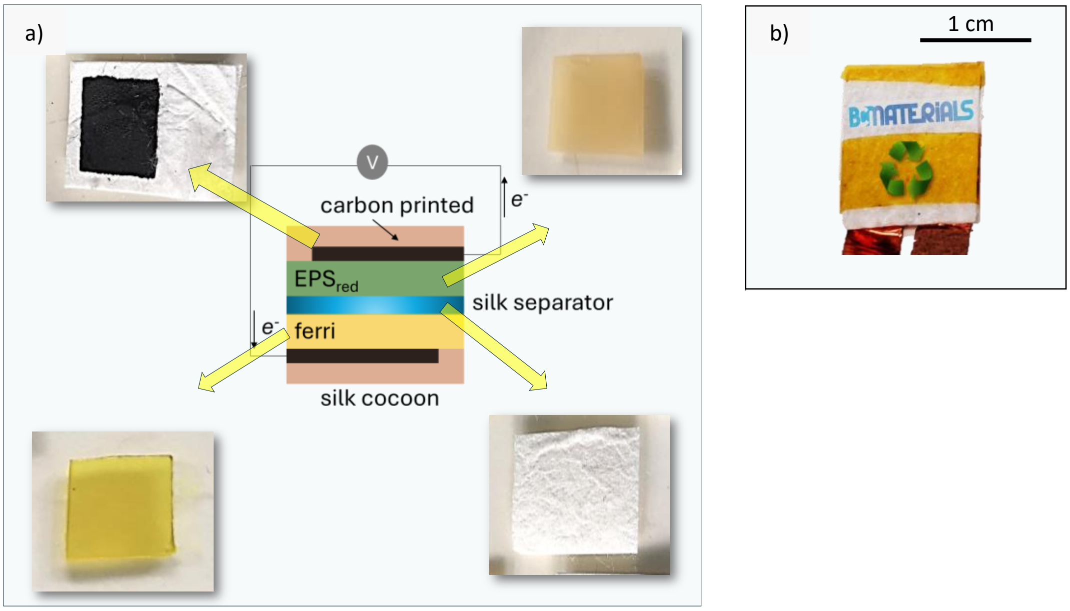

Supplementary Figure S1. a) Schematic representation of the developed micro biobattery. Photographic picture of the different battery components: carbon electrodes printed over silk cocoon, silk cocoon separator, ferri embedded into fibroin film (cathode), and EPS embedded fibroin film (anode). **b)** Single biobattery module photograph.

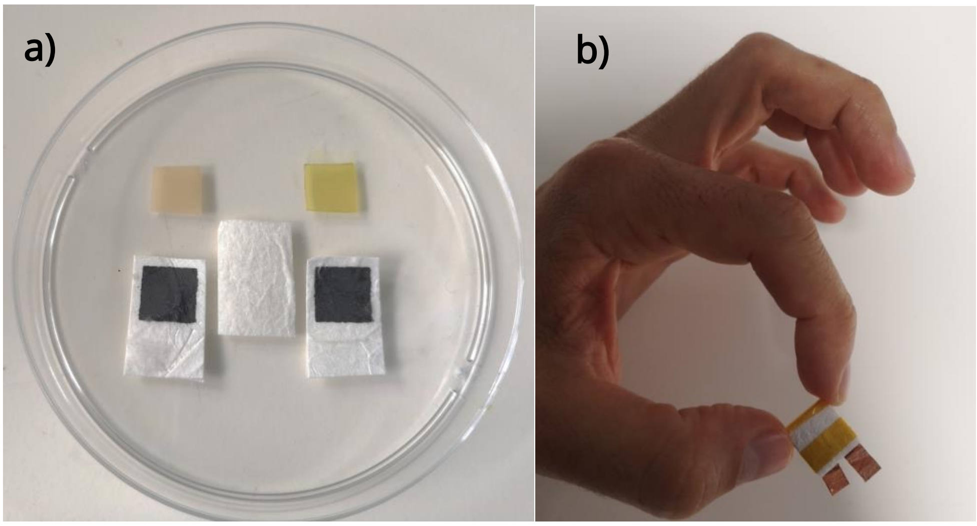

Supplementary Figure S2. Pictures of the fabricated biobattery: a) biobattery separated components and b) fabricated biobattery.

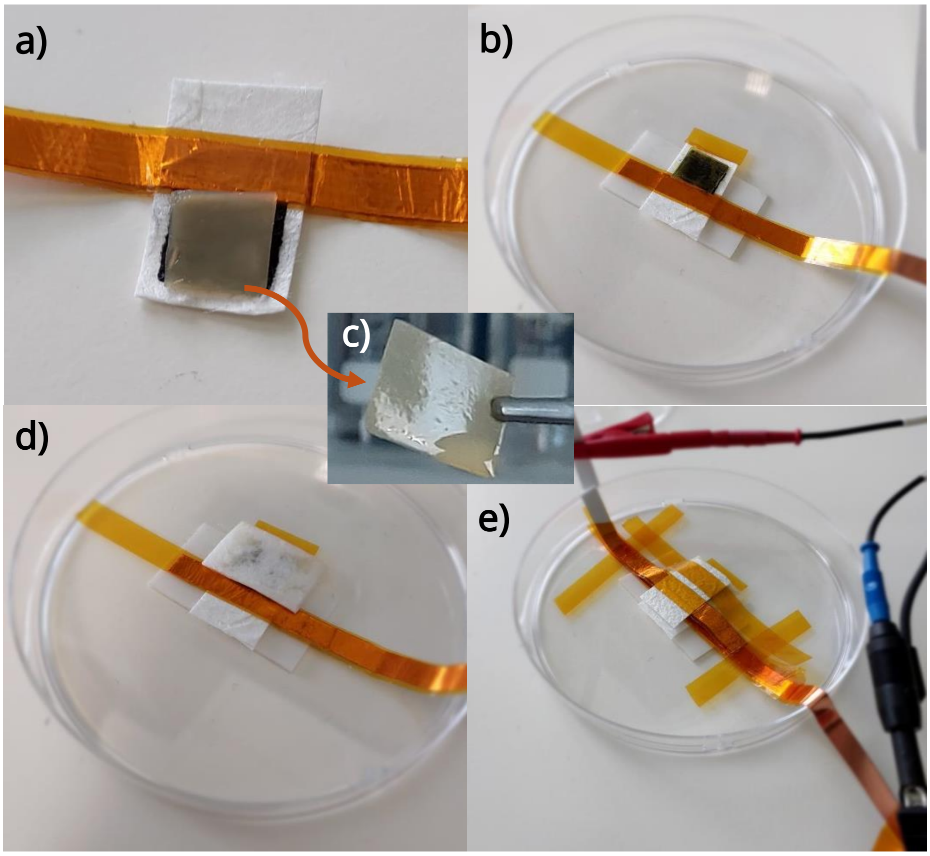

Supplementary Figure S3. Pictures of biobattery stacking. a) EPS_{red} adsorbed in silk fibroin film as anode over the top electrode of the biobattery. Copper tape is used for contacts, isolated from the chemical reactions with kapton tape. b) Silk fibroin film with adsorbed hexacyanoferrate solution as biocathode, over the carbon electrode. c) Amplified image of the bioanode. d) Pristine cocoon separator over the biocathode and e) final stack of the fabricated biobattery connected to the potentiostat for electrochemical measurements.

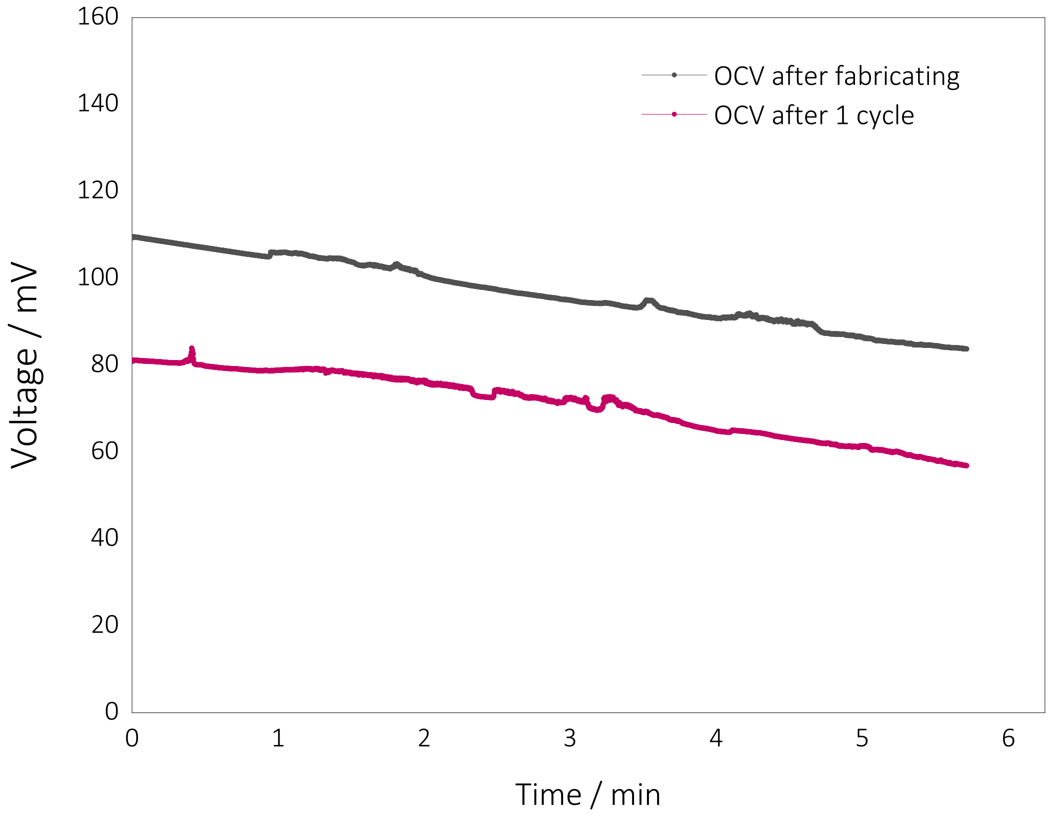

Supplementary Figure S4. Measured OCV using Whatman separator after fabricating the biobattery and after the first charge/discharge cycle.

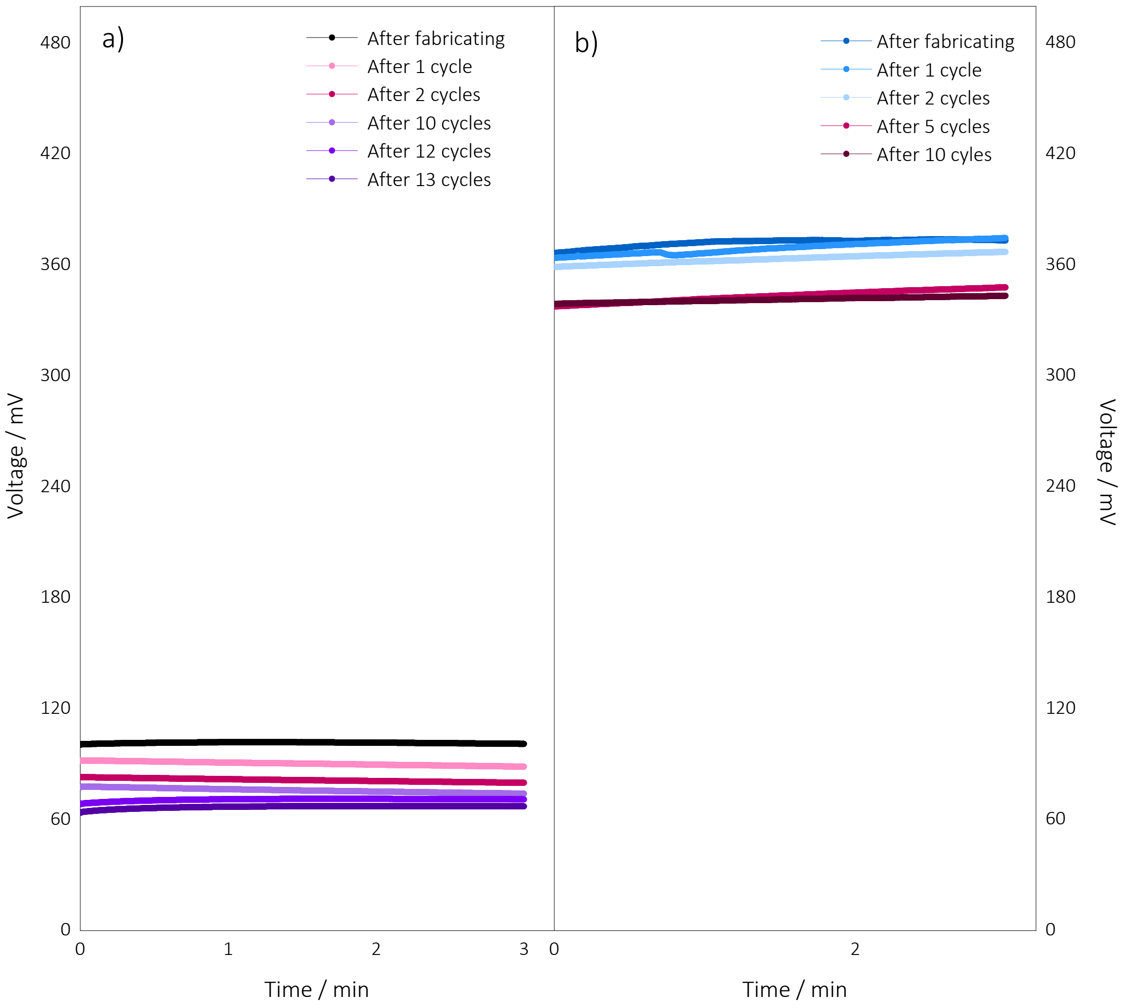

Supplementary Figure S5. OCV dependence on the number of charge/discharge cycles using a) pristine cocoon and b) O-silk separators.

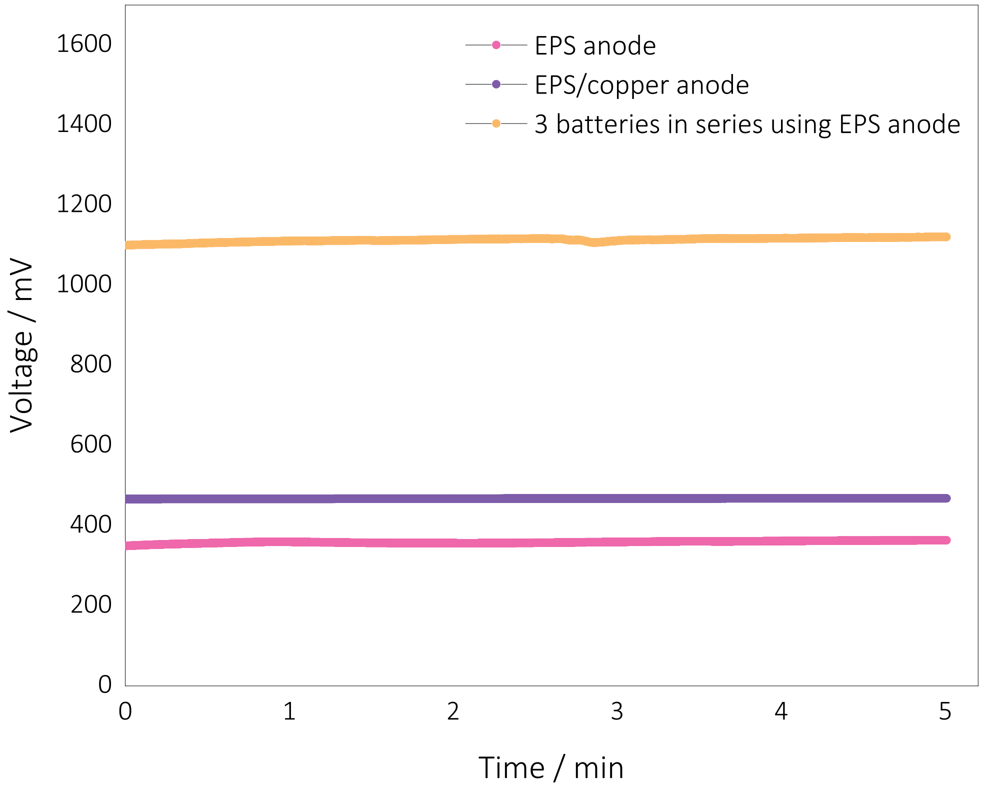

Supplementary Figure S6. OCV measurements using O-silk separator for different anodes and for three batteries in series using EPS as bioanode.

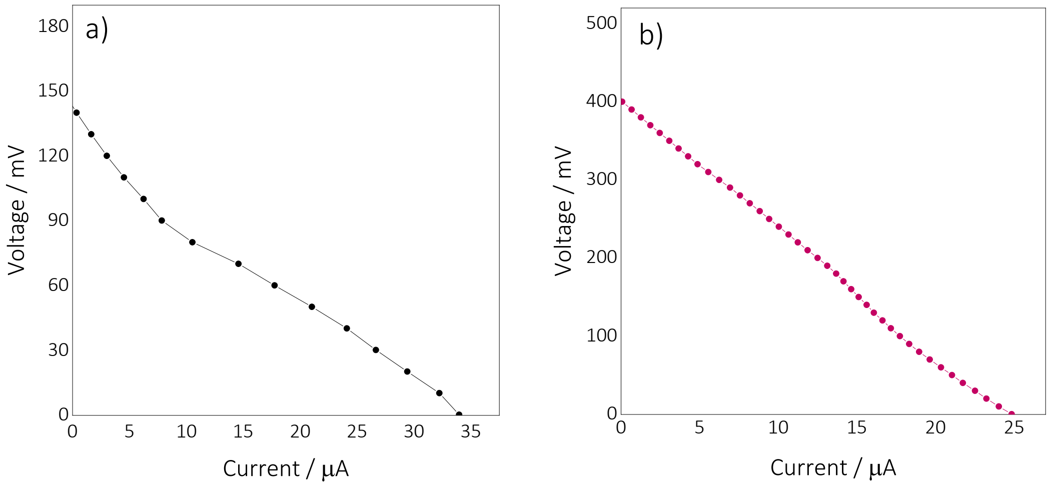

Supplementary Figure S7. Polarization curves using a) Whatman and b) pristine fiber separators.

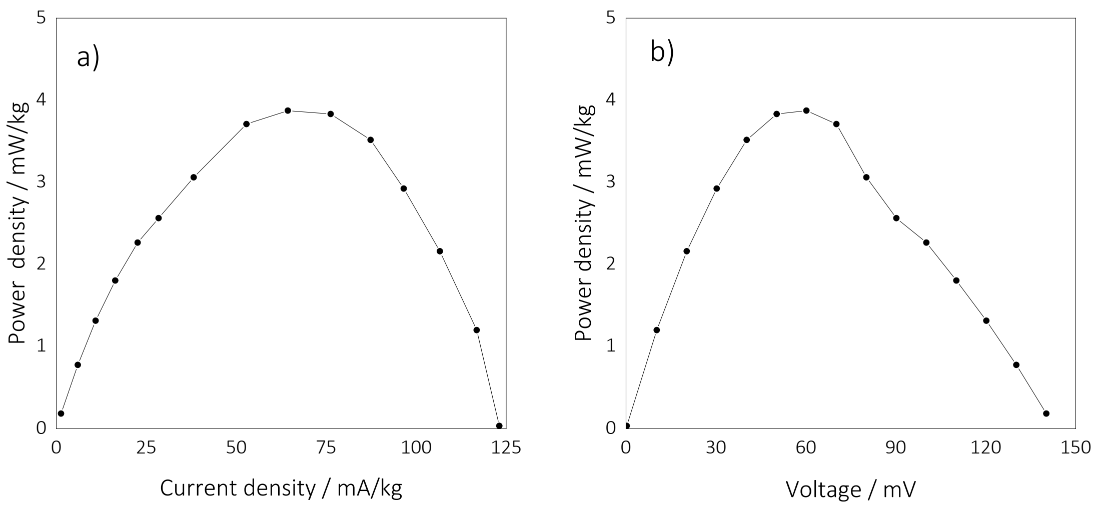

Supplementary Figure S8. Power curves using Whatman membrane. Power curve dependence on a) current density and b) voltage for biobatteries using Whatman membrane.

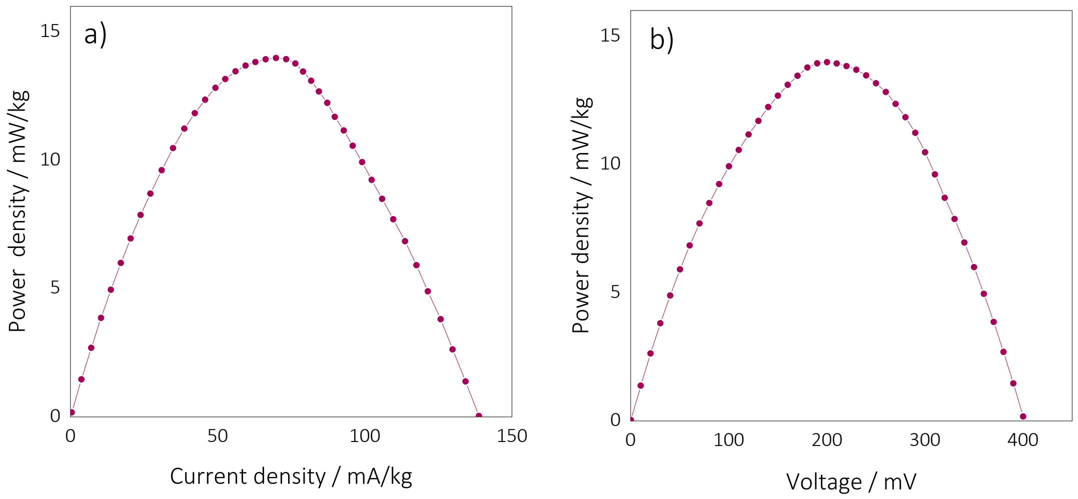

Supplementary Figure S9. Power curves using pristine fiber separator. Power curve dependence on a) current density and b) applied voltage.

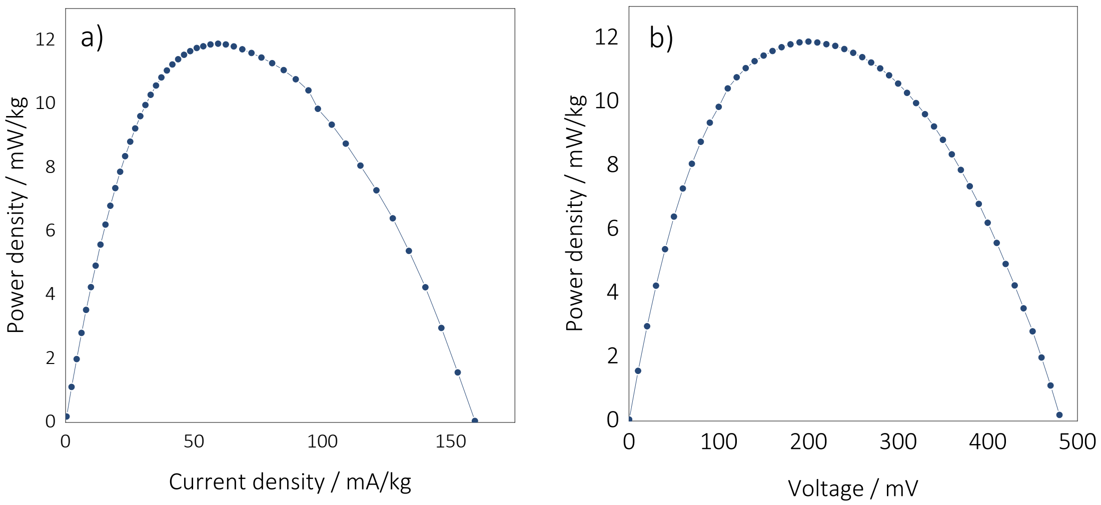

Supplementary Figure S10. Power curves using O-silk separator**.** Power curve dependence on a) current density and b) applied voltage.

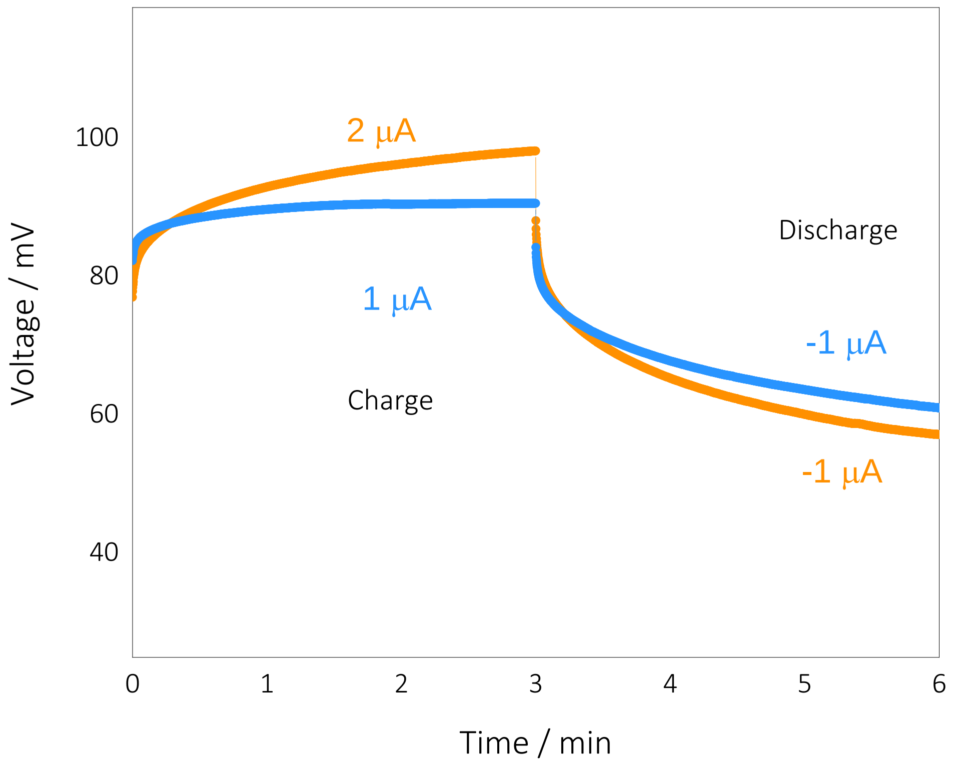

Supplementary Figure S11. Galvanostatic charge/discharge curves at different currents using pristine cocoon separator. Applied constant current of 2 and -2 μA (yellow line) and 1 and -1 μA (blue line).

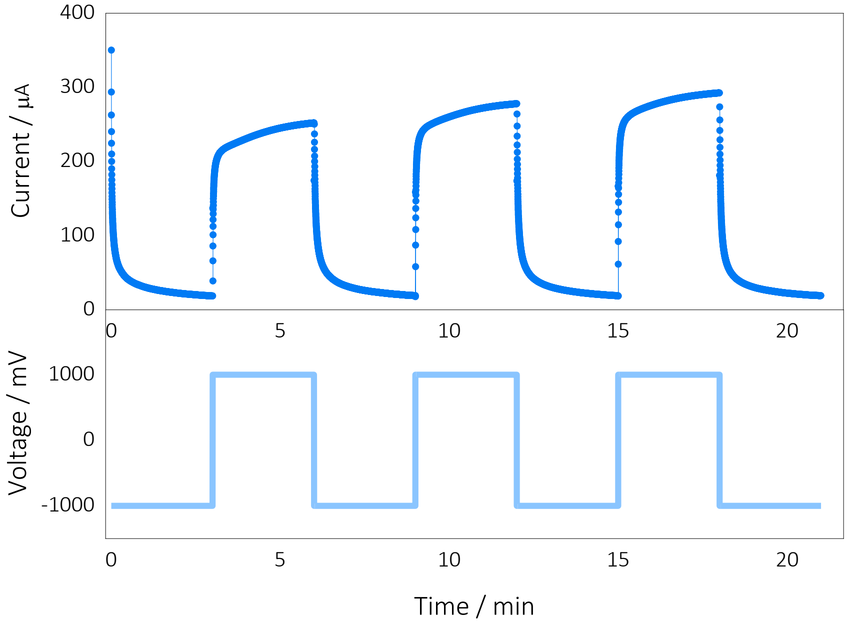

Supplementary Figure S12. Voltage constant charge/discharge cycles (dark blue line), applying 1 and -1 V (light blue line) using pristine fiber separator.

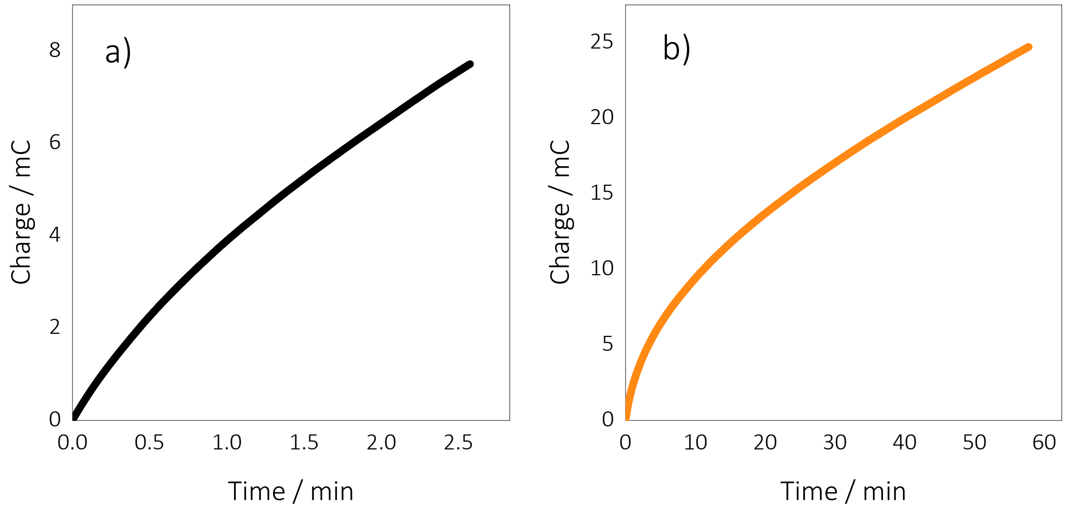

Supplementary Figure S13. Chronocoulometric measurements. Charge curve of biobattery using a) pristine cocoon and b) O-silk separator.

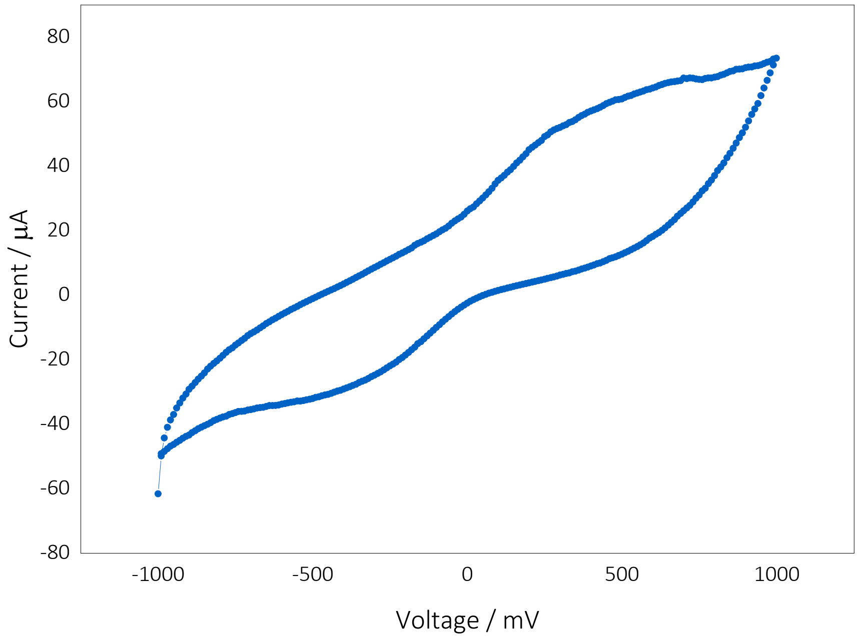

Supplementary Figure S14. Cyclic Voltammetry Measurements. Cyclic Voltammetry results of EPS biobattery using pristine fiber separator.

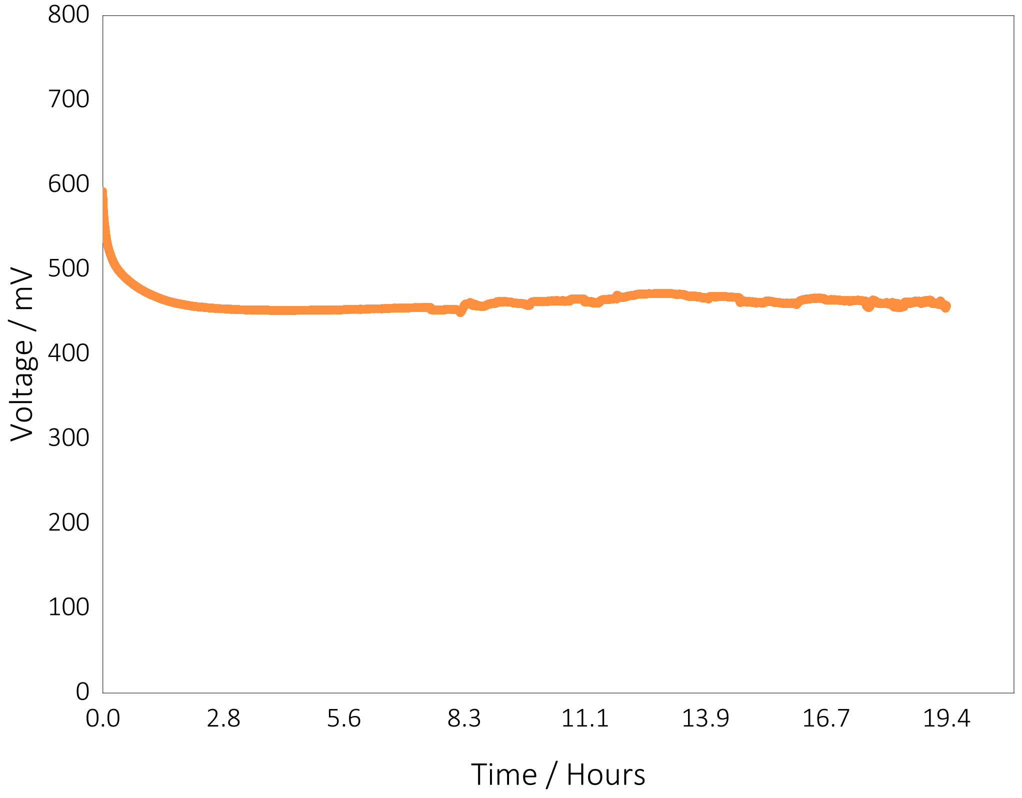

Supplementary Figure S15. Long galvanostatic discharge curve with 1 μA using EPS/copper as anode for 19.4 hours.

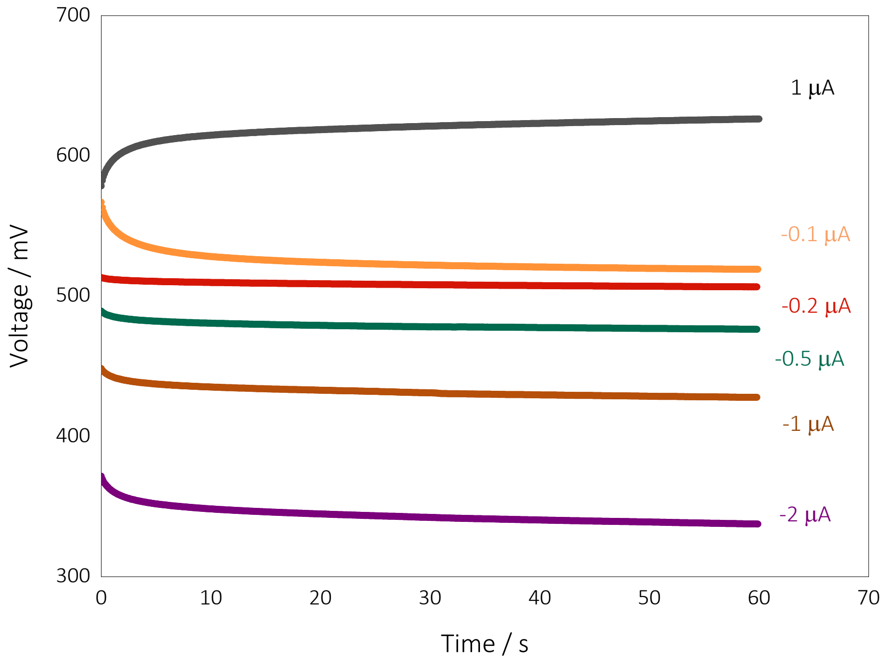

Supplementary Figure S16. Galvanostatic charge/discharge curves, using different discharge current values with EPS/copper anode. Charge with 1 µA (black line) and discharge using 0.1µA (yellow line), 0.2 µA (red line), 0.5 µA (green line), 1µA (brown line), and -2 µA (purple line).

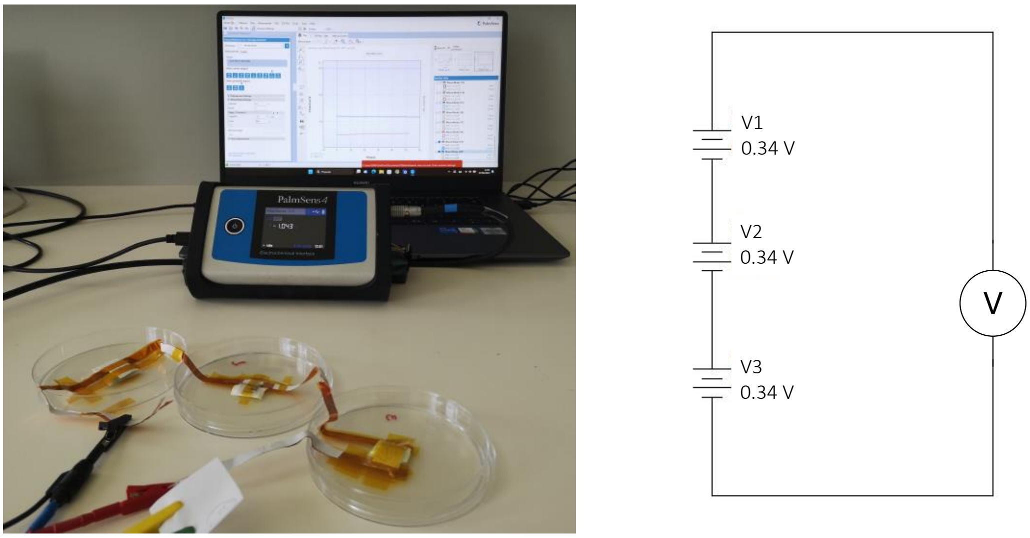

Supplementary Figure S17. a) Electrochemical measurements of three biobatteries in series. b) Electrical circuit of three biobatteries in series connected to a voltmeter.

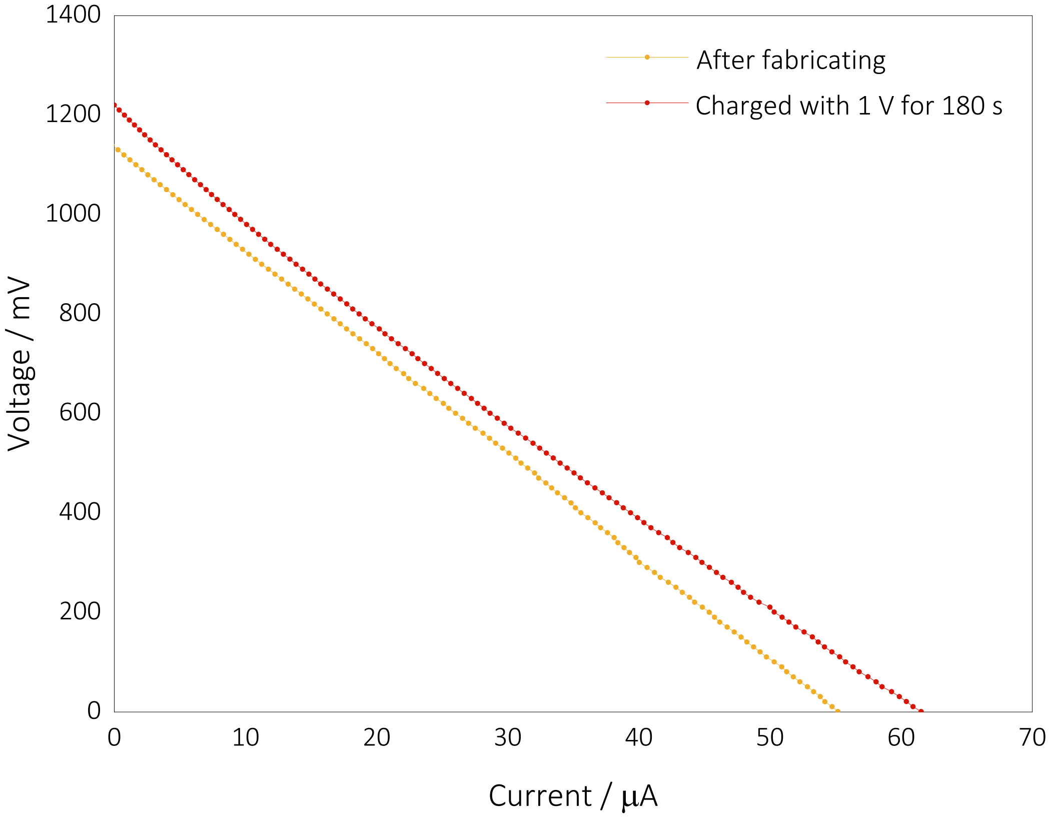

Supplementary Figure S18. Polarization curves of three biobatteries in series using O-silk separator and EPS bioanode.

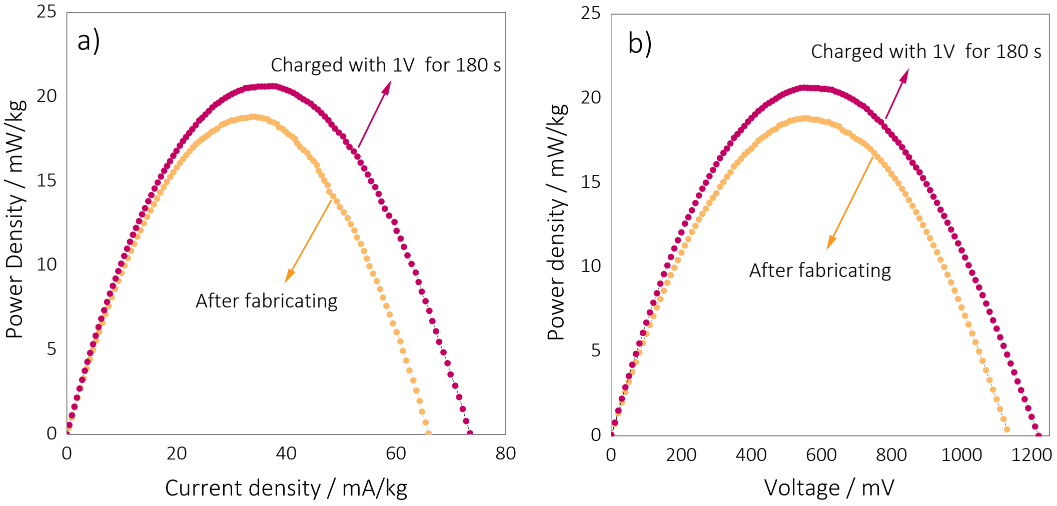

Supplementary Figure S19. Power curve of three batteries in series using O-silk separator and EPS bioanode. Power curve dependence on a) current density and b) applied voltage.

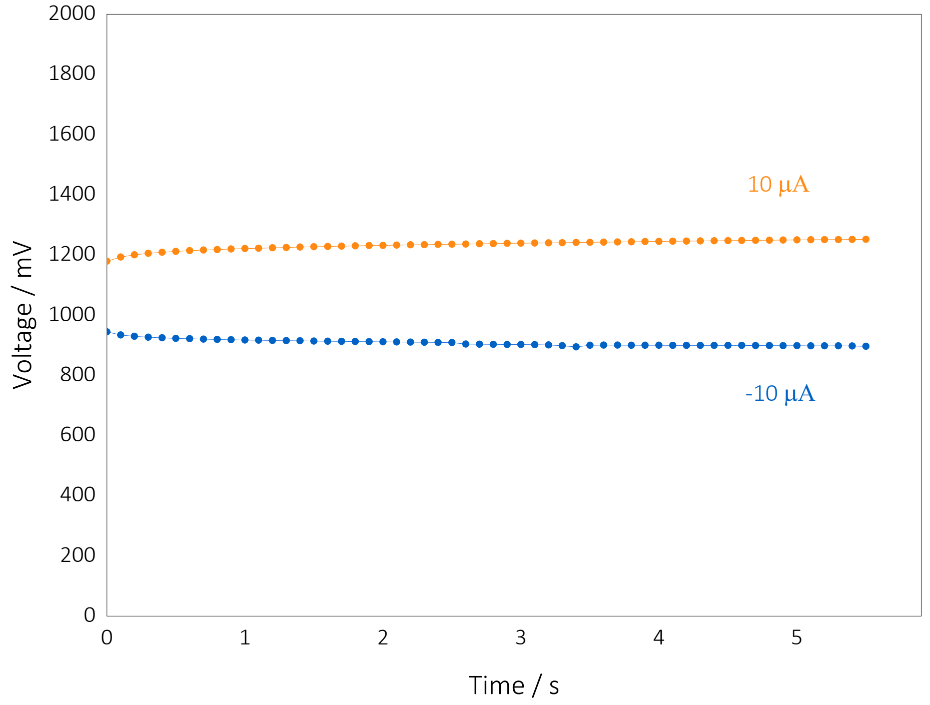

Supplementary Figure S20. Charge/discharge curves of three biobatteries in series using O-silk separator and EPS bioanode. Charge with 10 µA (yellow line) and discharge with 10 µA.
